# M. De Mignot on the Employment of Belladonna in the Treatment of Phimosis and Paraphimosis

**Published:** 1844-01-27

**Authors:** 


### M. DE MIGNOT ON THE EMPLOYMENT OF BELLADONNA IN THE TREATMENT OF PHIMOSIS AND PARAPHIMOSIS.

M. De Mignot, having derived benefit from the application of an ointment of belladonna in cases of phimosis and paraphimosis, recommends its employment in every case before having recourse to the knife. The ointment is made in the proportion of twelve grains of the extract of belladonna to thirty grains of simple cerate, and with this the prepuce is rubbed every hour. The dilating power of the belladonna soon begins to act, and in many cases an operation may be avoided. When the inflammation is violent, and the pain intense, he recommends to add a little opium and mucilage of quince seeds.—*Med. Gaz., from L'Experience.*

